# Enhancing the linear flow of fine granules through the addition of elongated particles

**DOI:** 10.1038/srep16071

**Published:** 2015-11-09

**Authors:** Zhiguo Guo, Xueli Chen, Yang Xu, Haifeng Liu

**Affiliations:** 1 Key Laboratory of Coal Gasification and Energy Chemical Engineering of Ministry of Education, East China University of Science and Technology, P. O. Box 272, Shanghai 200237, PR. China; 2 Shanghai Engineering Research Center of Coal Gasification, East China University of Science and Technology, P. O. Box 272, Shanghai 200237, PR. China

## Abstract

Sandglasses have been used to record time for thousands of years because of their constant flow rates; however, they now are drawing attention for their substantial scientific importance and extensive industrial applications. The presence of elongated particles in a binary granular system is believed to result in undesired flow because their shape implies a larger resistance to flow. However, our experiments demonstrate that the addition of elongated particles can substantially reduce the flow fluctuation of fine granules and produce a stable linear flow similar to that in an hourglass. On the basis of experimental data and previous reports of flow dynamics, we observed that the linear flow is driven by the “needle particle effect,” including flow orientation, reduced agglomeration, and local perturbation. This phenomenon is observed in several binary granular systems, including fine granules and secondary elongated particles, which demonstrates that our simple method can be widely applied to the accurate measurement of granular flows in industry.

A sandglass is a timing instrument that relies on the constant flow of sand^1^. These devices have been investigated for thousands of years, providing a convenient system to gain specific insights into fundamental scientific issues of granular flow behavior. In line with the poem that states, “to see a world in a grain of sand…”^2^, granular materials are important in various processes and display many peculiar intrinsic phenomena^3^,^4^,^5^,^6^. In the process of granular investigations over the past two centuries, the classic phenomena^7^,^8^ and typical experimental works^9^,^10^ have been mainly related to coarse and cohesionless particles whose diameters are larger than several hundred micrometers. However, mounting evidence^11^ indicates that more attention should be paid to the widespread application of fine granules in almost every aspect of our lives. For example, fine granular linear flows such as that of an hourglass are used in food production, chemical engineering, and pharmaceutical manufacturing to directly control the quality of the resulting product.

Nonetheless, granular flow remains a complicated scientific problem. Numerous unresolved flow mechanisms require extensive investigation^12^,^13^,^14^. The oscillatory phenomena reported in the sand hourglass have been confirmed by experiment and numerical simulations^15^,^16^ and are attributed to air-solid interactions and spontaneous sand organization. The flow fluctuations related to flow parameters such as porosity, coordination number, velocity magnitude, and stress^17^ have been quantitatively studied by using a 2D discrete model, and three regimes in the hourglass^18^ have been obtained from investigations concerning the effect of an interstitial fluid on granular discharge. Vivanco *et al.*^19^ have ascribed velocity fluctuations to an intermittent network of arches and strong force chains. Strong interparticle forces naturally form an inhomogeneous distribution of threadlike force chains^20^,^21^. Furthermore, force chains that propagate along a string of particles are likely to form an arch that can resist a certain pressure and prevent solid movement^22^,^23^. With respect to the jamming transitions of a granular system, Majmudar^24^ and Valverde^25^ have observed a power law behavior between the stress and the volume fraction and have reported that the granular jamming probability decreases with increasing ratio between the outlet diameter and particle size^26^.

Aeration and vibration^27^ have been demonstrated to improve fine granular flow. Common belief would suggest that the presence of needle-like particles in a binary granular mixture could negatively affect the flowability because needle-like shapes exhibit a high mobility resistance factor. In this letter, we report that the addition of needle-like particles to a fine powder gives rise to a linear flow that is analogous to that of an hourglass; we also systematically elucidate the transition mechanism. This counterintuitive result provides us an opportunity to carry out pioneering work in this area.

## Results

### Transition from intermittent flow to linear flow

**Figure 1:**
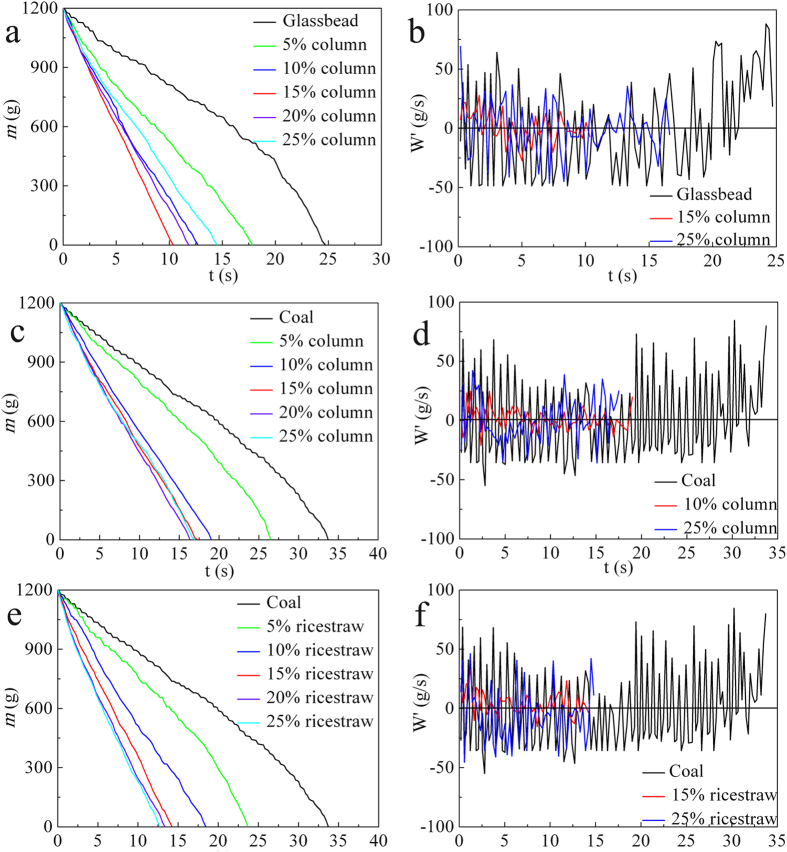
The flow behavior curves of different binary granular systems from a D_0_ = 32 mm aperture. (**a**) The loss weight curves of glass bead-columnar particle blend, (**b**) The fluctuation velocity of glass bead-columnar particle blend, (**c**) The loss weight curves of coal-columnar particle blend, (**d**) The fluctuation velocity of coal-columnar particle blend, (**e**) The loss weight curves of coal-rice straw blends, (**f**) The fluctuation velocity of coal-rice straw blend.

**Figure 2:**
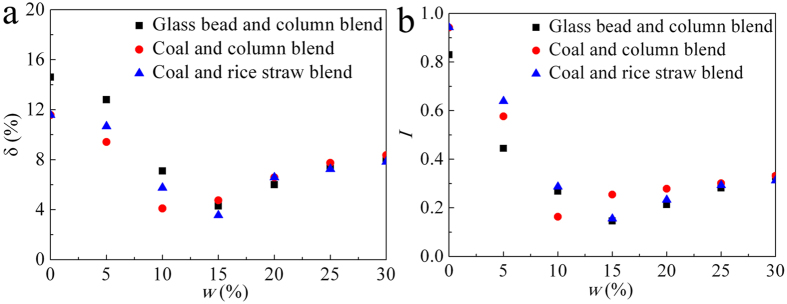
The fluctuation parameters as a function of needle particle mass fraction. (**a**) *δ* vs *w*, (**b**) *I* vs *w*.

For different binary granular systems, the weight-loss curves are plotted as a function of the elongated particle mass fraction (*w*) in Fig. 1a,c,e; the shape of the curves gradually transitions from terrace-like to linear with increasing *w*, and the best linear flow is observed at approximately 10–15 wt%. The occurrence of discontinuous flow indicates that a periodic time exists during the flow. There are zero velocity points for many cases that correspond to the arching state^1^. In contrast to the case of a fluid, the flow rate is independent of the bed height, except for the last few centimeters. The addition of needle particles improves the linearity of the weight-loss curve during the whole discharge process. As *w* increases, the vibration amplitude and sequential periodicity decrease (Fig. 1b). The time series of the fluctuating flow rate in Fig. 1 shows that a low-magnitude fluctuation velocity and linear weightlessness curve are obtained with the increase of *w*. In Fig. 2a, the granular flow degree of linearity (*δ*) decreases rapidly upon the addition of rice straw, which corresponds to the transition of the flow from instability to continuous, corresponding to its weightlessness curve. In addition, the trend of the fluctuation intensity (*I*) is similar to that of *δ*, both of whose minimum values are in the approximate range of 10 to 15%. This result means that the granular flow under the optimized addition amount is analogous to that in an hourglass, which is often used as a method to measure the passage of time. The decreased fluctuation also indicates steady granular flow.

Interestingly, we find that the phenomenon is observed for several binary granular systems, which allows us to extract constitutive relations. A detailed comparison demonstrates that the addition of needle particles to a cohesive powder enhances the linear flow dynamics. This observation may be a universal phenomenon.

### Experimental verification: Needle particle effect

The addition of elongated particles to a fine powder positively affects the linear flow of the fine particles, and the best linear flow is observed at an elongated-particle content of approximately 10–15 wt%. This phenomenon is referred to as the “needle particle effect” on the basis of the observation and the needle-like shape of the secondary particles. From microscopic to macroscopic granular systems, we theoretically and experimentally analyze the mechanism of the needle particle effect on the basis of the decreased agglomeration, needle particle orientation and macroscopic flow pattern.

**Figure 3:**
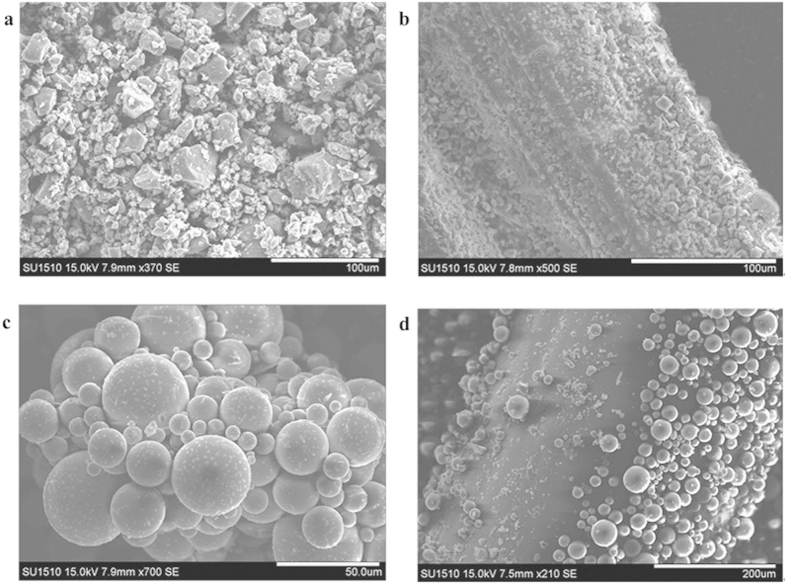
Images of agglomeration and adhesion states. (**a**) Pulverized coal, (**b**) fine coal adhere on the rice straw surface, (**c**) glass bead, (**d**) fine glass bead adhere on the columnar particle.

**Figure 4:**
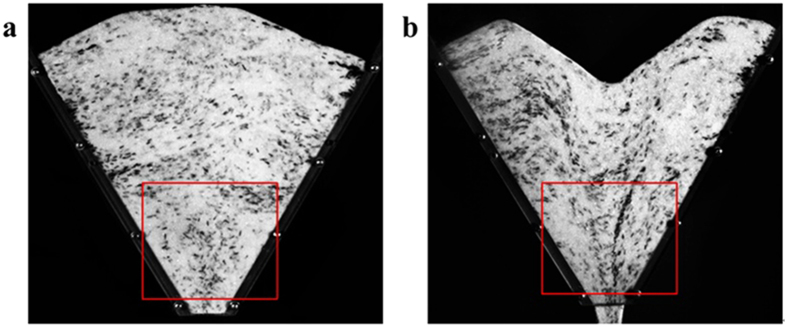
The orientation of needle particle in the evolution of granular flow pattern. (**a**) at the stationary state, (**b**) flow state after 0.5 second.

With an increase in *w*, the fine particles will adhere to the needle particles because of interparticle forces (Fig. 3). The interparticle cohesion is reduced upon the formation of large particle, which, in turn, leads to a decrease in the contact forces. Meanwhile, the needle particles flow in a uniform vertical orientation, in line with the flow direction near the hopper outlet, avoiding the arch due to mechanical interactions (Fig. 4). On the microscropic level, the flow fluctuation of the cohesive powder is mainly controlled by an intermittent network of arches and complex force chains^28^. Because of their large aspect ratio and orientation, needle particles possess a perturbation length, and their back-and-forth vibration could make the stress transmission more uniform^21^. We argue that the transmission of stress arises from a combination of uniform orientation and effective packing. Because of their needle-like shape, the particles behave as fibers that are widely spread throughout the binary granular system. Each needle particle is analogous to a stress magnet that attracts more fine particles because of its similar stress direction. Therefore, the role of needle particles in a fine powder system is analogous to the role of a policeman dealing with a traffic jam.

Our work reveals another surprising observation: the flow is enhanced in the range 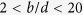, where *b* is the width of the needle particles and *d* is the diameter of the host particles. As the needle particle diameter decreases, it decreases the perturbation surface and intensifies the complexity force chain, thus increasing the flow resistance force. For large needle particles, the flow granular system should comply with the criterion 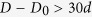 (where *D* is the inner diameter of the hopper and *D*_0_ is the outlet diameter) because of the dimensional effect.

**Figure 5:**
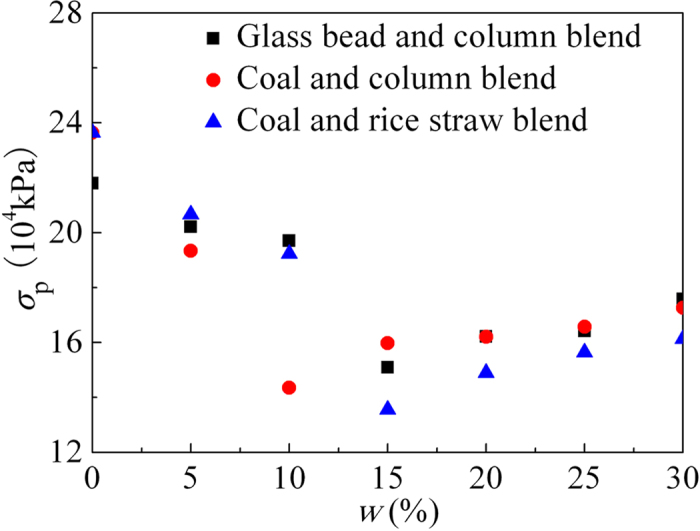
*σ*_p_ as a function of *w*.

To quantitatively characterize the transition of the flow pattern, the agglomeration strength (*σ*_p_) is introduced to indicate the degree of clustering; *σ*_p_ is defined as 
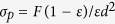
, where *ε* is the porosity of the granular system, *F* is interparticle force, and *d* is coal particle diameter. Notably, *d* is the granular diameter after adhesion due to the vertical orientation and small number of needle particles. In our previous work, we deduced the following equation: 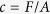, where *c* is the cohesion strength, which can be measured by using an FT-4 powder rheometer, and *A* is the cross-sectional area of a horizontal section. Thus, *σ*_p_ can be expressed as 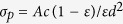. Here, the decrease in *σ*_p_ is mainly attributed to the penetration and adhesion of elongated particles. When the mass fraction exceeds a certain value, both the cohesion and diameter increase because of the increased mechanical interactions. Thus, the combined effect produces a small change in *σ*_p_. The quantitative results in Fig. 5 are consistent with the previous discussion of the flow behavior.

**Figure 6:**
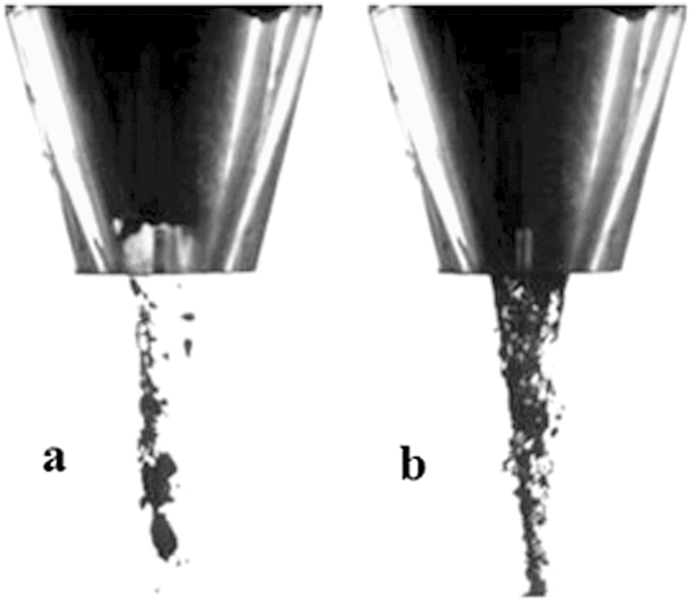
The comparison between arch and continuum flow for different samples. (**a**) Pulverized coal, (**b**) Coal- rice straw blend (15 wt% rice straw).

The predominant mechanism resulting in the unstable discharge of cohesive powder is a multi-alternative arching stage and break-arching stage; i.e., the presence of oscillating flow is produced by instability arches. Figure 6 exhibits an overview of this phenomenon. Because of its strong interparticle bonds, the fine powder is likely to form an arch near the hopper outlet, and this arch can sustain a certain stress. This arch may break because of its weight and because of the stress from the particles above. The oscillation process is the direct reason for the unstable flow rate, which corresponds to the results in Fig. 6a^17^,^19^. The addition of needle particles could effectively decrease the force intensity, thereby shattering the arch to generate continuous flow. When the concentration of needle particles is high, mechanical interactions may play a substantial role in resisting the granular flow.

## Discussion

The results of this study contradict the conventional belief that needle particles generate undesirable flow behavior because their large aspect ratio results in mechanical effects. Interestingly, the optimized addition of needle particles can effectively improve the flow behavior of fine powders, resulting in linear flow in the hopper. As described in ref. 3minute pressure fluctuations can tentatively lead to flow cessation in an hourglass. In an unconfined hopper, we expect that decreased agglomeration strength and flow orientation could be regulated by the linear flow of fine granules. The adhesion of fine particles and the needle particle vertical orientation produce a small cohesive force, which lessens the probability of the arching and jamming phenomena. The penetration of needle particles into the dense cohesive powder could further break the instability arch and result in stable flow. The decreased agglomeration strength provides intuitive evidence of the transition from intermittent flow to continuous flow. Hence, given our results and the evidence presented in the literature^27^, we conclude that the addition of needle particles can effectively improve the flow linearity of fine granules. On the basis of our previous research, when the *w* of the secondary needle particles exceeds an appropriate scope, the flow characteristics worsen because of strong mechanical effects. Thus, to obtain the required flow behavior, a moderate amount of secondary particles should be added.

In summary, the addition of a small amount of needle particles to a cohesive powder improves the linear flow, similarly to that in an hourglass. From a practical viewpoint, this work could be widely used in production applications because of the widespread use of granular flow and the importance of flowability. For the transition from intermittent flow to continuous flow, we have uncovered the dominant mechanism. Rather than aeration and mechanical methods, the addition of secondary particles improves the stable flow because of its inertness to chemical conversion. Most importantly, this approach could be applied in the manufacture of pharmaceuticals, which demands accurate measurements, opening a new approach to complete the granular theoretical framework.

## Methods

### Materials

To confirm the needle particle effect, we chose pulverized coal, fine glass beads, rice straw, and a column made of PVC. The Sauter diameters of the coal and glass beads were 22.1 μm and 17.9 μm, respectively. The aspect ratios of the rice straw and column were 4.31 and 5.39, and their widths were 325 μm and 395 μm, respectively. Because the moisture content is a key factor affecting the flow properties of a powder, the moisture content of the experimental material was controlled to be less than 2%, as measured by an MA150 infrared moisture meter.

### Experimental setup

**Figure 7:**
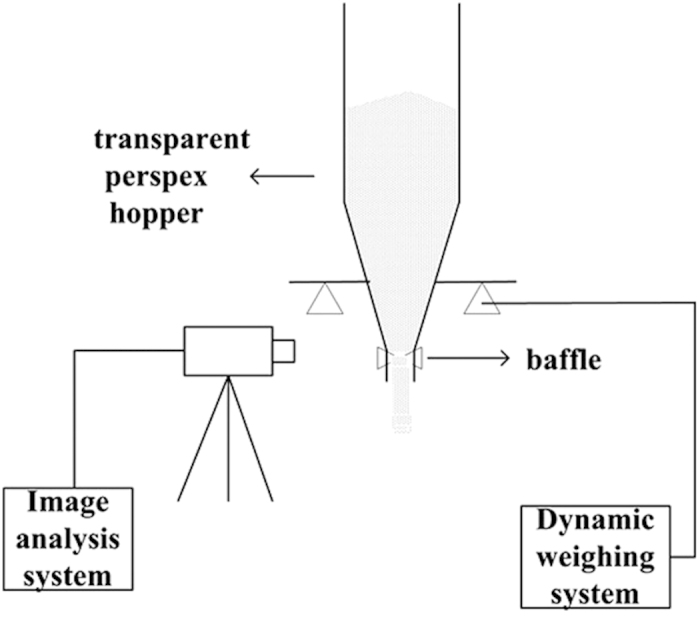
The schematic diagram of the experimental system.

The experimental system (Fig. 7) consisted of a transparent hopper, a weigh-sensor system, and a high-speed camera. The experiments were conducted on the premise that granular flow was steady and continuous. In our previous study, the hopper was demonstrated to satisfy the experimental standard through the investigation of gravity discharge. Its structural parameters were as follows: a column diameter of 150 mm, a cone with a half-opening angle of 15°, an outlet diameter of 32 mm, and a height of 220 mm. To capture the clear needle particle orientation, a two-dimensional hopper (width of 1.5 cm) and a high-speed camera were used. The width of the hopper was five times the longest length of the needle particles, which avoided the particle vertical orientation because of the dimensional effect. To analyze the stability of the discharge rate, an online dynamic weight system was used to record the hopper weight change. The data can be used to construct a variation-of-mass curve. Microimages of the blends were collected by a scanning electron microscope to confirm the occurrence of adhesion.

### Definitions of parameters

To accurately quantify the fluctuations in granular flow, the average flow rate 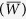 can be written 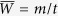, where *m* is the total weight of the samples and *t* is the flow time. The degree of linearity (*δ*) is defined as 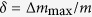, where Δ*m*_max_ is the maximum deviation between the instantaneous mass and the linear mass. The instantaneous velocity (*W*_t_) is defined as 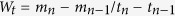 , where *m*_*i*_ and *t*_*i*_ are the instantaneous mass and time, respectively. The fluctuation velocity (*W*′) can be calculated by 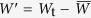. To quantify the flow stability, the fluctuation intensity (*I*) can be expressed as 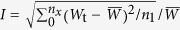.

### Cohesion measurements

Cohesion (*c*) corresponds to the intercept of the yield locus on the shear stress axis, which is often composed of the resistance from the mutual biting of granules and the united effect of condensation and colloids. It can be obtained after the shear test.

## Additional Information

**How to cite this article**: Guo, Z. *et al.* Enhancing the linear flow of fine granules through the addition of elongated particles. *Sci. Rep.* **5**, 16071; doi: 10.1038/srep16071 (2015).
